# Analysis tools to quantify dissemination of pathology in zebrafish larvae

**DOI:** 10.1038/s41598-020-59932-1

**Published:** 2020-02-21

**Authors:** David R. Stirling, Oniz Suleyman, Eliza Gil, Philip M. Elks, Vincenzo Torraca, Mahdad Noursadeghi, Gillian S. Tomlinson

**Affiliations:** 1 Infection and Immunity, University College London, Cruciform Building, Gower Street, London, WC1E 6BT United Kingdom; 2 The Bateson Centre, University of Sheffield, Firth Court, Western Bank, Sheffield, S10 2TN United Kingdom; 3 Department of Infection, Immunity and Cardiovascular Disease, University of Sheffield, Medical School, Beech Hill Road, Sheffield, S10 2RX United Kingdom; 4 Department of Immunology and Infection, London School of Hygiene and Tropical Medicine, Keppel Street, London, WC1E 7HT United Kingdom

**Keywords:** Fluorescence imaging, Software

## Abstract

We describe new open source software called QuantiFish for rapid quantitation of fluorescent foci in zebrafish larvae, to support infection research in this animal model. QuantiFish extends the conventional measurements of bacterial load and number of bacterial foci to include measures for dissemination of infection. These are represented by the proportions of bacteria between foci and their spatial distribution. We showcase these measures by comparison of intravenous and hindbrain routes of *Mycobacterium marinum* infection, which are indistinguishable by measurement of bacterial load and not consistently differentiated by the number of bacterial foci. The intravenous route showed dose dependent dissemination of infection, reflected by increased spatial dispersion of bacteria and lower proportions of bacteria distributed across many foci. In contrast, hindbrain infection resulted in localised disease, limited to a smaller area and higher proportions of bacteria distributed across fewer foci. The application of QuantiFish may extend beyond models of infection, to study other pathologies such as metastatic cancer.

## Introduction

Zebrafish are increasingly being used to address biological questions in the life sciences. Advantages of this model include genetic tractability, faithful representation of mammalian systems and optical transparency during the larval stages^1^. The availability of transgenic lines with fluorescent immune cell lineages and fluorescent protein-expressing microbes provide unparalleled opportunities for quantitative *in vivo* imaging, particularly in relation to host-pathogen interactions^2^. Currently, disease severity in zebrafish larval infection models is almost exclusively determined by quantitative imaging of fluorescent pathogens, by measuring total area or intensity of fluorescence as a surrogate for pathogen burden^3–25^. In the most established zebrafish larval infection model, using *Mycobcaterium marinum* (Mm) to model human tuberculosis (TB), other less commonly reported measures of severity include the total number of bacterial foci^3,16,23^, the number of bacteria per macrophage^3,11,12,24^, the presence of bacterial foci distal to the injection site^3,13,15,16^, survival^18,20,21,24–26^ and cording, indicating extracellular mycobacterial growth^12,18,26^. Although detection and quantitation of the number of fluorescent foci can be automated using image analysis software, the other parameters are determined manually, limiting their utility for high throughput and objective assessments (Supplementary Table S1). These approaches fail to consider pathogen dissemination as an alternative measure of disease severity. In order to address this deficiency, we have developed new, open source image analysis software called QuantiFish. This enables rapid and objective quantitation of pathogen load and of dissemination, using a range of parameters. We tested this software using Mm infection of zebrafish larvae and anticipate that it will also be applicable to other zebrafish models of infection and metastatic cancer.

## Results

### Comparison of QuantiFish and other image analysis software

To develop our analysis tools we used images of zebrafish larvae intravenously infected with Mm concordantly classified by two independent investigators as examples of minimally, moderately or widely disseminated infection (Supplementary Fig. S1). Images were analysed with QuantiFish, which provides a simple interface for rapid, sensitive quantitation of total fluorescence and signal from individual fluorescent foci, defined as a continuous region of fluorescence above a threshold set by the user (Supplementary Fig. S2). Our software represents a significant advance compared to existing programs to quantify fluorescence^19,24,27,28^, both because of its intuitive interface and more importantly, its ability to generate detailed information about the characteristics of each individual fluorescent focus, without the need for additional custom scripts (Supplementary Table S2). Total bacterial load (integrated fluorescence intensity) and numbers of separate foci of bacteria detected using QuantiFish were significantly higher in fish with more widely disseminated infection (Supplementary Fig. S3). We compared the performance of QuantiFish to that of a published ImageJ macro^24^, selected as an example of freely available software which does not rely on additional custom scripts, that has previously been used to quantify fluorescence in zebrafish larval Mm infection^10–12,18,20,24^. This generated comparable results which were very highly correlated with those obtained using QuantiFish (Supplementary Fig. S3).

### A need for analysis tools to quantify dissemination of pathology

**Figure 1:**
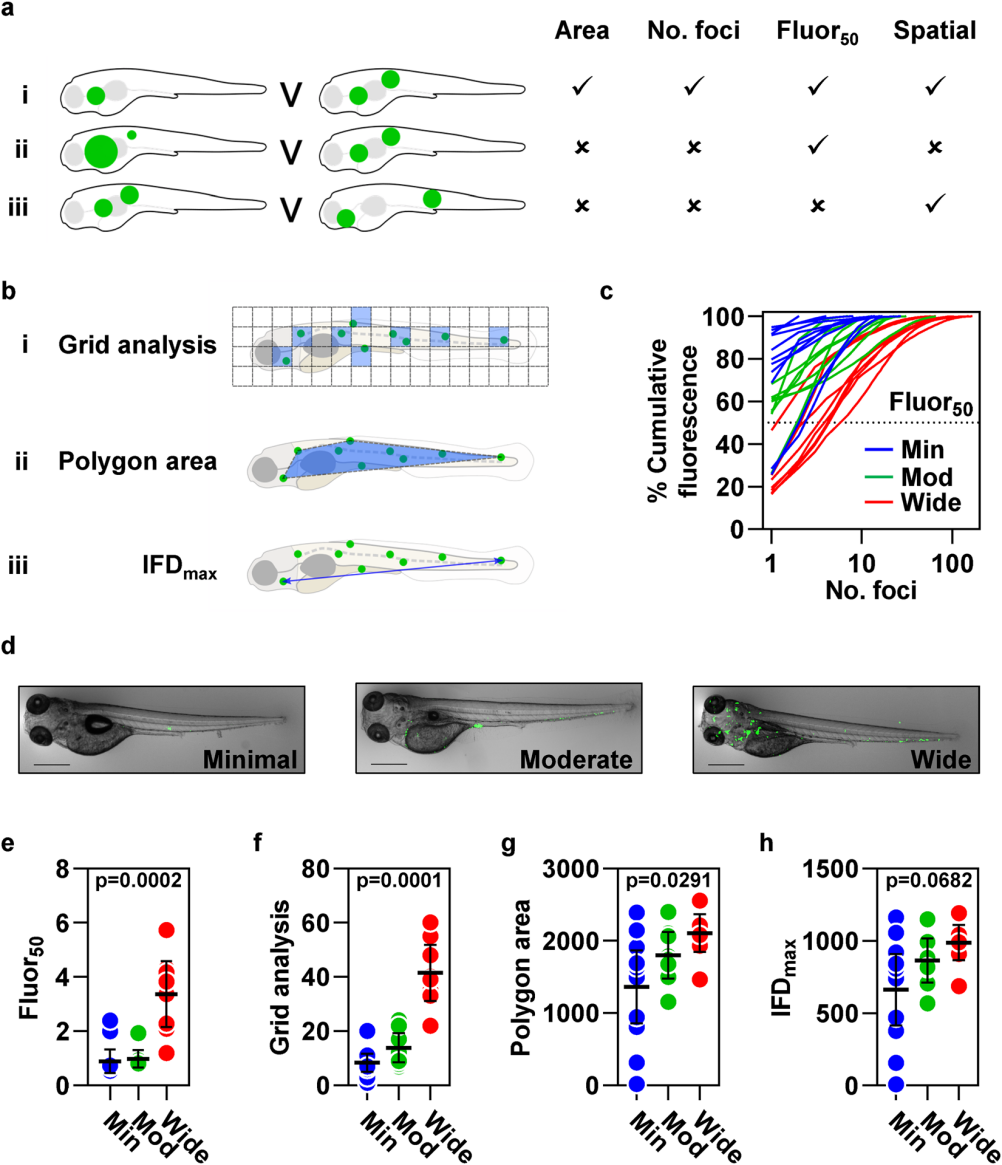
Analysis tools to quantify dissemination of mycobacterial infection. Limitations of existing outcome measurements of infection, illustrated in schematic diagrams representing different distributions of fluorescent bacterial foci (green). Area of fluorescent signal and number of foci distinguish some distributions of infection (**ai**) but not others (**aii–iii**). We propose fluor_50_, the number of foci that contribute 50% of the total fluorescence, to distinguish differences in the proportional distribution of the total burden of pathology at each site (**aii**) and parameters that quantify the spatial distribution of foci to differentiate localised dissemination from distant dissemination (**aiii**). (**bi**) Grid analysis divides the image into an array of squares, then quantifies the number of grid zones containing the centre point of ≥1 foci (highlighted blue). (**bii**) The area of a polygon (highlighted blue) encompassing the centre points of all foci. (**biii**) The maximum distance between the centre points of any two foci (IFD_max_). (**c**–**h**) Quantitation of bacterial dissemination in images of zebrafish larvae four days post intravenous infection with 200–400 cfu *Mycobacterium marinum* expressing mWasabi, selected from three independent experiments, classified as having minimally, moderately or widely disseminated infection (n = 11, 8 and 8, respectively). (**c**) The relationship between cumulative percentage fluorescence and the number of foci which generate this signal. (**d**) Representative images from the data presented in (**e**–**h**) of zebrafish larvae with each category of dissemination. Scale bars, 500 µm. (**e**) Fluor_50_, calculated by interpolation of the data shown in (**c**). Spatial measurements of dissemination, (**f**) grid analysis, (**g**) polygon area and (**h**) IFD_max_. Lines (**c**) and data points (**e**–**h**) represent individual zebrafish larvae. Lines and error bars (**e**–**h**) represent median ± IQR. p values were derived from Kruskal-Wallis tests with Benjamini, Krieger and Yekutieli correction for multiple comparisons.

We exploited the information from individual fluorescent foci generated by QuantiFish to develop new analysis tools to evaluate features of bacterial dissemination which are not possible to assess using existing strategies that measure area or intensity of bacterial fluorescence or count the total number of fluorescent foci without any regard to their spatial distribution^19,24,27,28^. We conceptualised the limitations of current automated image analysis methods in schematic representations of different distributions of fluorescent foci (Fig. 1a). The first example (Fig. 1ai) compares differences in the distribution of infection that are measured by current methods to quantify the total fluorescence or the number of fluorescent foci. However, neither measure distinguishes differences in the proportion of fluorescence in different foci, for example if 90% of the bacteria are contained within one of two foci, or equally distributed between two foci (Fig. 1aii). Nor can they discriminate localised dissemination of infection from distant dissemination of infection (Fig. 1aiii). To address these limitations, we propose four measures of bacterial dissemination. First, the number of fluorescent bacterial foci that are responsible for 50% of the total fluorescence; second the number of predefined grid zones that contain the centre point of a bacterial focus (Fig. 1bi); third the area of a polygon containing the centre points of all foci (Fig. 1bii) and fourth the maximum distance between any two foci (Fig. 1biii).

### Quantifying proportional distribution of pathology across disease sites

We reasoned that in less disseminated infection a higher proportion of the bacterial fluorescence would be localised in fewer foci. This was demonstrated by plotting the percentage cumulative fluorescence in each fish as a function of the number of fluorescent foci (Fig. 1c). A summary statistic for each fish was then derived by linear interpolation of these data to estimate from the known data points, the unknown data point, namely, the number of fluorescent foci responsible for 50% of the total fluorescence (fluor_50_). In some instances, fluor_50_ can be a non-integer, most simply illustrated by the example of a fish which only contains one focus, where the fluor_50_ value is 0.5. This measure was significantly different in groups of fish independently classified as having minimally, moderately or widely disseminated infection and distinguished widely disseminated infection from the other two groups (Fig. 1d,e).

### Parameters to measure spatial distribution of pathology

We also sought to develop strategies to assess the spatial distribution of fluorescent foci, on the premise that bacterial foci distributed over a broader area represent more highly disseminated infection, indicative of more severe disease. Our first approach, termed “grid analysis”, quantifies the number of user-defined grid zones that contain the centre point of one or more foci of fluorescence. Multiple fluorescent foci located within a single zone are counted as one positive grid section, emphasizing larger differences in spatial distribution than simply counting the number of foci (Fig. 1bi). Our second approach uses the co-ordinates that describe the locations of the centre points of individual fluorescent foci to compute the area of the smallest convex polygon that contains all these points (Fig. 1bii). Our third strategy extends this approach by using the points that define the boundaries of this polygon to calculate the maximum distance between any two fluorescent foci (IFD_max_) (Fig. 1biii). These three measures were each concordant with the investigators classification of the degree of dissemination. The spatial distribution of bacteria was highest in widely disseminated infection, intermediate in moderately disseminated infection and lowest in minimally disseminated infection. Both grid analysis and polygon area showed significant differences between minimally, moderately and widely disseminated infection (Fig. 1f–h).

### Inoculum dose determines burden and dissemination of pathology in intravenous infection

**Figure 2:**
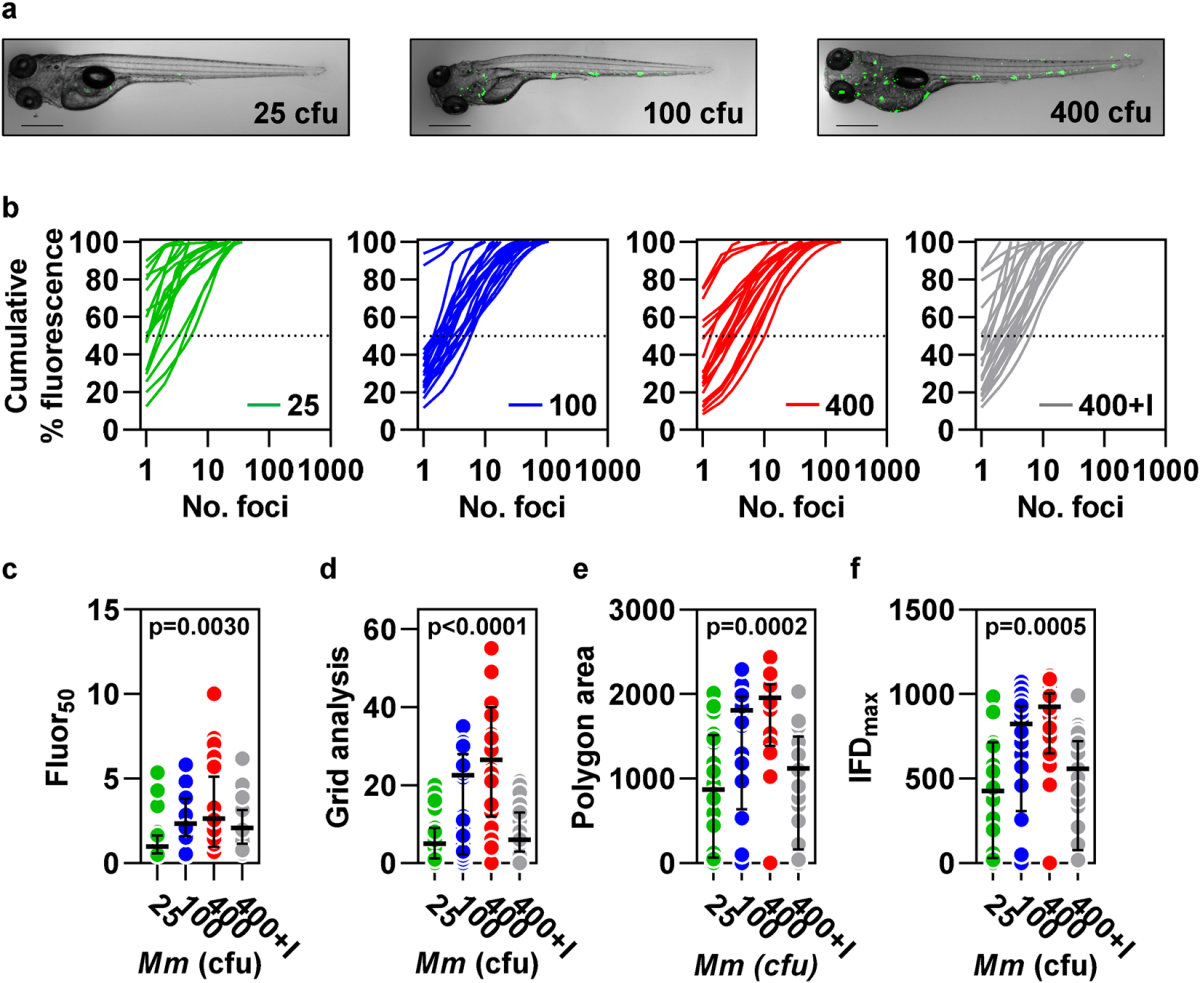
Quantitation of bacterial dissemination in response to a dose titration of intravenous *Mycobacterium marinum* (Mm) infection. Measures of dissemination in zebrafish larvae four days after intravenous infection with 25, 100 or 400 cfu Mm ± 400 µM isoniazid (I) (n = 24, 28, 22 and 25, respectively). (**a**) Representative images are shown for each inoculum dose. Scale bars, 500 µm. (**b**) The relationship between cumulative percentage fluorescence and the number of bacterial foci responsible for this signal. (**c**) Fluor_50_, calculated by interpolation of the data shown in (**b**). The three spatial dissemination parameters, (**d**) grid analysis, (**e**) polygon area and (**f**) IFD_max_. Lines (**b**) and data points (**c**–**f**) represent individual zebrafish larvae. Lines and error bars (**c**–**f**) represent median ± IQR. p values were derived from Kruskal-Wallis tests with Benjamini, Krieger and Yekutieli correction for multiple comparisons. Data are representative of three independent experiments.

We then tested the hypothesis that infection with higher doses of Mm would lead to greater dissemination. First, we demonstrated the expected incremental increase in bacterial burden in zebrafish larvae intravenously infected with a dose titration of Mm (25, 100 and 400 colony forming units (cfu)), using both QuantiFish and ImageJ, which provided highly correlated results (Supplementary Fig. S4). Treatment with the anti-mycobacterial drug, isoniazid, significantly diminished detected fluorescence, consistent with control of infection (Supplementary Fig. S4). Escalation of the inoculum dose also led to higher numbers of fluorescent foci, and treatment with isoniazid was associated with significantly fewer foci (Supplementary Fig. S4). Next, we applied our dissemination analysis tools to quantify the proportional distribution of the total bacterial load between sites and the spatial distribution of bacteria within each fish. Higher inoculum dose was associated with significantly increased dissemination, demonstrated by smaller proportions of the total fluorescence divided between larger numbers of foci (Fig. 2a–c) and by the three measurements that reflect the spatial distribution of bacteria (Fig. 2d–f). The spatial measures of dissemination consistently increased with escalation of the inoculum dose (Fig. 2d–f and Supplementary Fig. 5), but in some experiments with very high bacterial burden, fluor_50_ was lower in larvae infected with 400 cfu Mm than in those infected with 100 cfu Mm (Supplementary Fig. 5). This was due to bacteria which coalesced in long continuous strands in fish infected with the highest inoculum dose (Supplementary Fig. 5). We interpret these data as evidence that inoculum dose directly influences both the spatial dispersion of bacteria, and the ability of the host to predominantly contain bacterial growth to fewer sites, reflected by the fluor_50_ parameter.

### Dissemination parameters represent novel measures of disease severity

**Figure 3:**
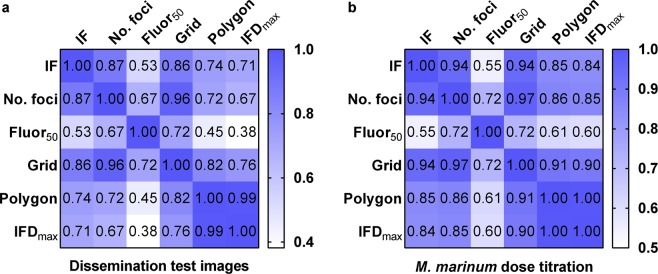
Correlation matrices of relationships between integrated fluorescence, number of foci and parameters of dissemination. Spearman rank correlation matrices of the associations between existing measurements of outcome of bacterial infection; integrated fluorescence (IF) and number of foci and our new measures of dissemination; fluor_50_, grid analysis (Grid), polygon area (Polygon) and IFD_max_ in (**a**) the images of *Mycobacterium marinum* (Mm) infected larvae used to develop our analysis tools (n = 27) and (**b**) the Mm dose titration experiment (n = 99). Data presented in (**a**) were derived from three independent experiments and (**b**) are representative of three independent experiments.

We next quantified the relationship between our dissemination parameters and existing outcome measurements, by Spearman rank correlation analysis (Fig. 3). None of our novel measures correlated perfectly with integrated fluorescence or the number of foci, which were highly correlated with each other. Fluor_50_, in particular, was less strongly associated with either of the existing measures of outcome compared to the other dissemination measurements. Grid analysis was highly correlated with the number of foci and polygon area was almost perfectly correlated with IFD_max_. This is in keeping with the strategies used to derive these parameters, where the second variable is related to the first. Overall, this analysis suggests that our parameters represent distinct measures of outcome that likely reflect different aspects of infection.

### Validation of dissemination analysis tools using a localised hindbrain infection model

**Figure 4:**
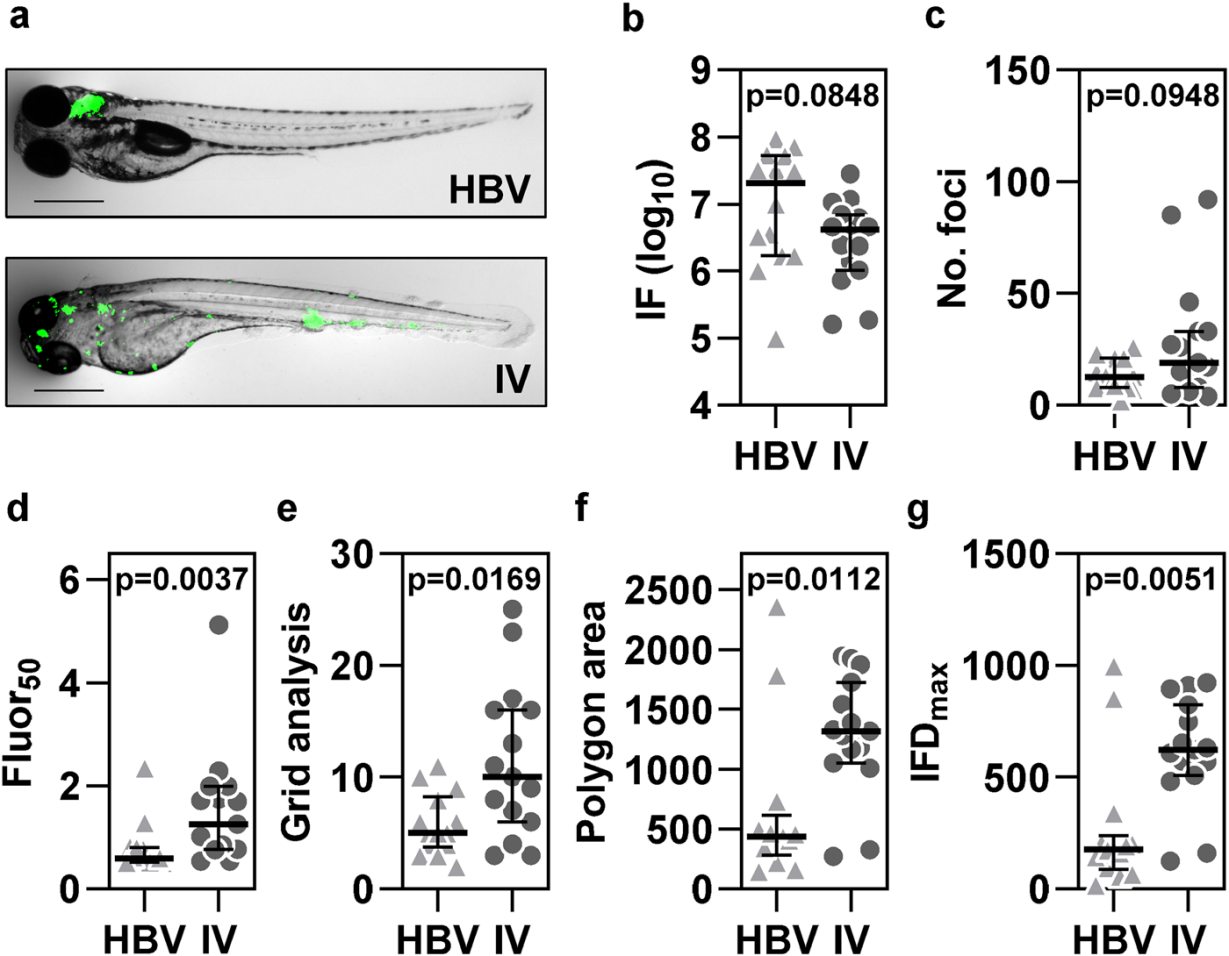
Comparison of localised and systemic *Mycobacterium marinum* (Mm) infection. (**a**) Representative images are shown for zebrafish larvae four days after either localised hindbrain ventricle (HBV) infection (n = 14) or systemic intravenous (IV) infection (n = 15) with 100 cfu Mm. Scale bars, 500 µm. Existing outcome measures, integrated fluorescence (IF), a surrogate for total bacterial burden (**b**) and the number of fluorescent bacterial foci (**c**) and dissemination parameters, fluor_50_ (**d**), grid analysis (**e**), polygon area (**f**) and IFD_max_ (**g**) are presented. Data points represent individual zebrafish larvae. Lines and error bars represent median ± IQR. p values were derived from Mann-Whitney tests. Data are representative of three independent experiments. See also Supplementary Fig. S7.

To further validate these analysis tools we used hindbrain ventricle (HBV) Mm infection as a model of localised pathology (Supplementary Fig. S6). Introduction of 100 cfu Mm into the HBV predominantly resulted in restriction of bacteria to the injection site (Fig. 4a, Supplementary Fig. S6). In comparison to embryos challenged intravenously with the same bacterial inoculum dose, as a model of widespread pathology, the most frequently used existing measure of disease severity, integrated fluorescence was equivalent (Fig. 4b, Supplementary Fig. S7). The number of fluorescent foci was also comparable for both infection routes in some experiments (Fig. 4c), although in others, there were significantly fewer foci in HBV infected embryos (Supplementary Fig. S7). By contrast, fluor_50_ was always significantly lower in the HBV infection model, consistent with our hypothesis that in less disseminated infection the majority of pathology would be contained to a few foci (Fig. 4d, Supplementary Fig. S7). The spatial measures of dissemination were invariably significantly higher in intravenous infection, with particularly marked differences evident for the polygon area and IFD_max_ (Fig. 4e–g, Supplementary Fig. S7). Taken together, these data indicate that as anticipated, haematogenous infection generates much more widely distributed pathology. Importantly, our findings highlight the additional resolution provided by our analysis tools to reliably elucidate differences between experimental groups that are not consistently detectable using currently available methods.

## Discussion

We have generated a suite of tools to quantify spatial distribution of pathology and the proportional distribution of the total disease burden between sites in zebrafish larvae. In the context of infection, these novel measurements of outcome provide greater capacity to detect differences following experimental manipulation, that might not otherwise be elucidated when principally reliant on pathogen burden as an outcome measure. This, in turn, could potentially provide new insights into immunological mechanisms of protection and pathogenesis or identify pathways for therapeutic intervention. We anticipate that our tools could also be applied to other zebrafish models where spatial distribution of focal pathology is important, such as metastatic cancer. As such, these analysis methods represent an exciting advance, of considerable utility to a wide range of investigators engaged in zebrafish research.

## Methods

### Zebrafish

Zebrafish were raised and maintained on a 14/10 light/dark cycle at 28.5 °C according to standard protocols^29^ in the Zebrafish Facility at University College London. Work was approved by the British Home Office (Project License 70/8900). All experiments were performed in accordance with the relevant guidelines and regulations, on larvae up to five days post fertilisation, before they are protected under the Animals (Scientific Procedures) Act. Adult AB/TL (wild type) zebrafish were spawned to generate embryos for infection experiments. Embryos were maintained at 28.5 °C in egg water containing 60 µg/ml Tropic Marin Sea Salt (Norwood Aquarium) and anaesthetised with egg water containing 200 µg/ml buffered tricaine (3-aminobenzoic acid ethyl ester) (Sigma-Aldrich) during bacterial injections and imaging. Egg water was supplemented with 0.003% PTU (1-phenyl-2-thiourea) (Sigma-Aldrich) after bacterial injections had been performed, in order to inhibit melanisation.

### M. marinum infection

*Mycobacterium marinum* (Mm) M strain expressing the pmsp12 mWasabi^24^ or psMT3 mCherry^23^ vector was cultured on Middlebrook 7H10 agar (Becton Dickinson and Company) supplemented with 0.5% oleic acid/albumin/dextrose/catalase (OADC) (Becton Dickinson and Company), 0.5% glycerol (Sigma-Aldrich) and hygromycin (50 µg/ml) (Fisher Scientific). To generate injection inocula, Mm from agar plates was cultured statically at 28.5 °C for 24 hours in Middlebrook 7H9 broth (Becton Dickinson and Company) supplemented with 10% albumin/dextrose/catalase (ADC) (Becton Dickinson and Company) and hygromycin (50 µg/ml) and harvested at mid-log growth (optical density (OD)_600nm_ 0.7–1), (OD)_600nm_ 1 representing 10^8^ colony forming units (cfu)/ml. Harvested bacteria were washed three times in phosphate buffered saline (PBS) (Gibco) and resuspended in 2% polyvinylpyrrolidone (PVP)40 (Sigma-Aldrich)/PBS containing 10% phenol red (Sigma-Aldrich) to aid visualisation of injections^30^.

Zebrafish embryos staged at 24 hours post-fertilisation (hpf) were manually dechorionated using jeweller’s forceps (Dumont #5, World Precision Instruments), then infected at 28–30 hpf by injection of Mm into the caudal vein or the hindbrain ventricle (HBV)^30^. In three independent experiments from which images were used to develop our dissemination analysis tools, embryos were intravenously infected with 200–400 cfu Mm in a volume of 1 nl. For the Mm dose titration experiment, larvae were intravenously infected with 1 nl of bacterial suspension containing 25, 100 or 400 cfu. A subset of larvae infected with 400 cfu Mm were maintained in egg water containing 400 µM isoniazid (Sigma-Aldrich) to control infection^25^. To generate localised infection, 100 cfu Mm was injected into the HBV in a volume of 1 nl.

### Stereofluorescence microscopy

Live, anaesthetised larvae were imaged four days post-infection (dpi) on a flat agarose plate, using an M205FA stereofluorescence microscope (Leica) with a 1x objective. Brightfield and fluorescence images were captured using a DFC365 FX camera (Leica) and exported as 8- or 16- bit TIF files for analysis.

### Classification of images used to develop analysis tools

To develop our analysis tools we used images of Mm infected zebrafish larvae in which fluorescence was represented as a binary channel to limit the visual impact of variable signal intensity. Images that were concordantly classified by subjective visual evaluation as representative of minimally, moderately or widely disseminated infection, by two independent investigators blinded to the experimental groups, were included.

### Image analysis

Images were analysed using QuantiFish and a published ImageJ macro^24^ to quantify bacterial burden and number of bacterial foci. Analysis tools to quantify dissemination of bacterial infection were developed in Python 3, then integrated into QuantiFish, which was used to quantify dissemination of bacterial infection.

### QuantiFish

QuantiFish is an open source application written in Python 3 and released under the GNU General Public License (version 3). Compiled installers are available for Windows and Mac, and the source code can be run on other operating systems. QuantiFish utilises the SciPy (http://www.scipy.org), NumPy^31^, Pillow (https://github.com/python-pillow), and scikit-image (skimage)^32^ libraries. We exploited existing packages for image processing within scikit-image and SciPy’s inbuilt spatial and mathematical functions. NumPy was selected because of its ability to perform operations on large data arrays more rapidly than the native Python functions^31^. The software provides an intuitive graphical user interface to enable automated analysis of fluorescence in zebrafish embryos. Images of individual fish in Tagged Image Format (.tif) are imported using the Python Imaging Library (Pillow fork) before being converted into NumPy arrays, which enables efficient manipulation by considering the image as an array of numbers. Values below the user-defined threshold are then removed to allow quantitation of the number of positive pixels and integrated fluorescence (the sum of all positive pixels) as surrogate measures of the total burden of pathology (Table 1).

**Table 1:** QuantiFish workflow and programming.

Workflow	Steps	Measurement Name	Package Functions Used
Load Image	Open image		PIL Image Class
	Convert to array		numpy.array
Basic Statistics	Fetch minimum and maximum intensity values	Minimum, Maximum	numpy.min, numpy.max
	Apply user-defined *threshold*, set pixels below threshold to 0		
	Count positive pixels	Positive Pixels	numpy.count_nonzero
	Calculate integrated intensity	Integrated Intensity	numpy.sum
Find Foci	Quantify local maxima (points of peak intensity)	Total Peaks	skimage.feature.peak_local_max
	Assign ID labels to confluent objects		skimage.measure.label
	Quantify the number of pixels associated with each object	Total Foci	numpy.unique
	Filter foci list based on *minimum size* set by user		
	Count objects larger than the size filter	Large Foci	numpy.sum
	Exclude staining in foci below size limit		
	Quantify pixels in positive foci	Positive Pixels in Large Foci	numpy.count_nonzero
	Quantify integrated intensity within positive foci	Integrated Intensity in Large Foci	numpy.sum
	Count local maxima in filtered foci	Peaks in Large Foci	skimage.feature.peak_local_max
Foci Statistics	Construct an array of statistics for each focus	Foci Area, Average, Max, Min and Integ. Intensity	skimage.measure.regionprops
	Record stats for each focus larger than the *minimum size*		
	Generate list of centroid coordinates for large foci		
Fluor_50_	Sort the focus statistics list by integrated intensity		
	Calculate percent fluorescence contributed by each object	Foci Percent Intensity	
	Calculate cumulative percent fluorescence	Foci Cumulative Percent Intensity	numpy.cumsum
	Determine fluor_50_ by linear interpolation	Fluor_50_	scipy.interpolate.interp1d
Spatial Statistics	Create blank array the size of the original image		numpy.zeros
	Plot foci centroid coordinates on blank array		
	Split array into grid segments of user-defined *size*		numpy.array_split
	Consider segment positive if any centroids are present		numpy.max
	Count positive and total segments	Total Grid Boxes, Positive Grid Boxes	
	Generate convex polygon using centroid coordinates	Foci Polygon Area	scipy.spatial.ConvexHull
	Select centroids which generate the polygon		scipy.spatial.ConvexHull.vertices
	Construct a distance matrix between selected points		scipy.spatial.distance_matrix
	Find maximum distance between foci from matrix	IFD_max_	scipy.spatial.distance.euclidean

The workflow and package functions used to generate the measurements produced by QuantiFish.

### Dissemination analysis tools

To generate the dissemination measurements, detailed information from each individual area of fluorescent signal, termed a “focus”, must first be collected (Table 1). Foci are detected by using scikit-image functions to identify pixels representing the peak intensity of an area of fluorescence and label continuous regions of signal above the desired threshold. The NumPy “unique” function is then used to evaluate the size of each labelled region before objects smaller than the user-defined minimum are excluded from further analysis. Objects are not further segmented due to the limited ability to separate touching features using a 2D image of a 3D embryo, alongside the limited resolution of images of entire fish, which prohibits visualisation of individual bacteria. Detected regions therefore represent foci of infection rather than individual cells or bacteria. The scikit-image “region properties” function is used to calculate and store an array of statistics for each region of fluorescence. The area, centre coordinates, minimum, maximum and mean intensity for each individual focus are extracted from the “region properties” array and optionally logged into a separate output file. Integrated intensity for each focus is calculated by multiplying the area of the focus by the mean intensity.

### Fluor_50_

To derive the fluor_50_ statistic, the integrated intensity of foci in each image is ranked largest to smallest, the percentage of the total fluorescence within each focus is calculated, then the cumulative percentage intensity is determined using the NumPy “cumulative sum” function. This is plotted against the number of foci responsible for the signal, then the number of objects that contribute 50% of the total fluorescence (fluor_50_) is estimated by linear interpolation using SciPy interpolation classes.

### Spatial distribution analysis parameters

For the spatial analyses, individual foci are considered as single points (centroids) to minimise skewing of data from unusually large objects. A list of focus centroid coordinates is extracted from the previously obtained “region properties” for each object and entered into an empty Boolean array in the shape of the original image, creating a “map” of centroids.

#### Grid analysis

To perform the “grid analysis” NumPy’s “array splitting” function divides the centroid map into squares of a size specified by the user. The algorithm classifies positive grid zones as those that contain coordinates for the centroid of any focus (designated “True” in the Boolean array), then quantifies the number of positive zones (as well as the total number of grid sections).

#### Polygon area

The list of focus centroid coordinates is also used to generate the “polygon area” parameter. We used the SciPy “ConvexHull” class to calculate the area of a polygon which encompasses the centroids of all foci, known as a convex hull.

#### Maximum inter-focus distance (IFD_max_)

Using the SciPy “Euclidean distance” function, the subset of points used to construct the polygon edges and vertices are taken forward to generate a distance matrix between all possible pairs of these centroids only, from which the two most widely separated are used to calculate the maximum inter-focus distance (IFD_max_).

#### Remark 1

Given that the centroids used to construct the polygon boundaries will always contain the two most distant points, restricting this analysis to these pairs of coordinates minimises the computational power needed to determine the IFD_max_. This is particularly relevant in images with large numbers of foci, for which analysis of all possible coordinate pairs would require substantially increased computational time.

#### Remark 2

It should be noted that a polygon cannot be generated in images containing fewer than three foci or where coordinates align to produce a 1D line with an area of zero. In these scenarios the software defaults to evaluation of all possible coordinate pairs to determine IFD_max_. However, it is extremely unlikely that images containing large numbers of foci would fail to produce a 2D polygon, hence the low probability of evaluating excessive numbers of irrelevant coordinate pairs that would lead to significantly increased computational time.

### Output files

Results are written to two .csv files, one containing summary statistics for each image and a second, optional file containing data for each individual region of signal. Key settings such as the threshold used and the fluorescence channel analysed (in multi-channel images) are also logged.

### ImageJ macro

Images were also analysed using a published ImageJ macro as previously described^24^. This generates output data for the number of foci (“Count”), area of signal (“Total Area”), the percentage of the total area occupied by detected signal (“%Area”) and the average size of foci (“Average Size”). The number of positive pixels was calculated by multiplying “%Area” by the total number of pixels for each image.

### Comparison of QuantiFish and ImageJ

QuantiFish offers several significant improvements on existing methods to analyse fluorescence in zebrafish embryos. Most notably, in addition to the currently used statistics of integrated fluorescence (or pixel counts) and object counts, this software provides new measurements for the spatial dispersion of foci and for the distribution of pathology across sites, as a proportion of the total disease burden. These parameters were successfully applied to quantify dissemination of infection, providing novel measurements of outcome that we anticipate will have significantly greater resolution to detect differences compared to existing tools.

The well-established NumPy and scikit libraries^31,32^ were used for efficient manipulation of image data, ensuring that the analysis can be implemented rapidly, with minimal user input, no requirement for additional scripts and without the need for dedicated hardware. The program utilises an intuitive user interface, with features such as automatic image bit depth detection which allows quantitation to be performed without the need for significant prior experience in image analysis. The program also supports selective filtering of files for analysis, eliminating the need for images to be manually sorted prior to quantitation. The inclusion of a previewing feature allows the user to visually inspect detected fluorescence in real time while configuring detection parameters, which facilitates the process of determining an appropriate threshold for a given data set. Overall, this software introduces novel dimensions for the analysis of fluorescence dissemination in zebrafish larvae while providing a simple and convenient interface which is accessible across the field.

### Statistical analyses

Statistical analysis was performed using GraphPad Prism 8 software. Kruskal-Wallis tests with the Benjamini, Krieger and Yekutieli correction method for multiple comparisons were used to analyse data from experiments which contained more than two groups. Mann-Whitney tests were used to analyse data from experiments that comprised two groups. Spearman rank correlation matrices were generated in GraphPad Prism 8.

## Supplementary information

Supplementary information.

## Data Availability

The data presented in this study are available from the corresponding author on request.

## Code availability

The source code and manual are available at https://github.com/DavidStirling/QuantiFish. The version of QuantiFish used in this paper is available as Supplementary Software.
