# Supplementary material for: Analysis tools to quantify dissemination of pathology in zebrafish larvae
Source: Sci Rep. 2020 Feb 21;10:3149. doi: 10.1038/s41598-020-59932-1 (PMC7035342; doi:10.1038/s41598-020-59932-1)
Supplement: Supplementary file 1 — Supplementary information. [file 41598_2020_59932_MOESM1_ESM.pdf]

SUPPLEMENTARY INFORMATION

TITLE

Analysis tools to quantify dissemination of pathology in zebrafish larvae

AUTHORS

David R. Stirling[1], Oniz Suleyman[1], Eliza Gil[1], Philip M. Elks[2,3], Vincenzo Torraca[4], Mahdad Noursadeghi[1], Gillian S. Tomlinson[1]*

AFFILIATIONS

[1]Infection and Immunity, University College London, Cruciform Building, Gower Street, London WC1E 6BT, United Kingdom. [2]The Bateson Centre and [3]Department of Infection, Immunity and Cardiovascular Disease, University of Sheffield, Firth Court, Western Bank, Sheffield S10 2TN, United Kingdom. [4]Department of Immunology and Infection, London School of Hygiene and Tropical Medicine, Keppel Street, London WC1E 7HT, United Kingdom.

CORRESPONDING AUTHOR

*Dr Gillian Tomlinson, Infection and Immunity, University College London, Cruciform Building, Gower Street, London WC1E 6BT. Email: g.tomlinson@ucl.ac.uk, ORCID ID: 0000-0003-4342-3161.

	Total fluorescence	No. foci	Bacteria per macrophage	Distal foci	Survival	Cording
Fluorescence imaging required	✓	✓	✓	✓	×	×
Automated	✓	✓	×	×	×	×
Rapid	✓	✓	×	×	×	×
Objective	✓	✓	×	×	✓	×
Continuous scale	✓	✓	×	×	×	×

Supplementary Table S1: Existing measures of disease severity in zebrafish larval *Mycobacterium marinum* infection.

Software	Free	Custom script / protocol	GUI	User friendly	Manual file processing	Detection preview	Total fluorescence	No. foci	$Fluor_{50}$	Grid	Polygon	IFD_{max}
QuantiFish	✓	×	✓	✓	×	✓	✓	✓	✓	✓	✓	✓
ImageJ (Takaki)[1]	✓	×	×	×	✓	×	✓	✓	×	×	×	×
ImageJ (other)[2]	✓	×	×	×	✓	×	✓	✓	×	×	×	×
Delphi[3]	×	×	✓	✓	✓	×	✓	×	×	×	×	×
Volocity[4]*	×	✓	✓	×	×	✓	✓	✓	×	?	?	?

Supplementary Table S2: Comparison of software used to quantify fluorescence in zebrafish larval *Mycobacterium marinum* infection.

*Information that was possible to obtain without a license is provided for this proprietary programme.

REFERENCES

1. Takaki, K., Davis, J. M., Winglee, K. & Ramakrishnan, L. Evaluation of the pathogenesis and treatment of Mycobacterium marinum infection in zebrafish. *Nat. Protoc.* **8**, 1114–1124 (2013).
2. Matty, M. A., Oehlers, S. H. & Tobin, D. M. Live Imaging of Host-Pathogen Interactions in Zebrafish Larvae. *Methods Mol. Biol. Clifton NJ* **1451**, 207–223 (2016).
3. Stoop, E. J. M. *et al.* Zebrafish embryo screen for mycobacterial genes involved in the initiation of granuloma formation reveals a newly identified ESX-1 component. *Dis. Model. Mech.* **4**, 526–536 (2011).
4. Schiebler, M. *et al.* Functional drug screening reveals anticonvulsants as enhancers of mTOR-independent autophagic killing of Mycobacterium tuberculosis through inositol depletion. *EMBO Mol. Med.* **7**, 127–139 (2015).

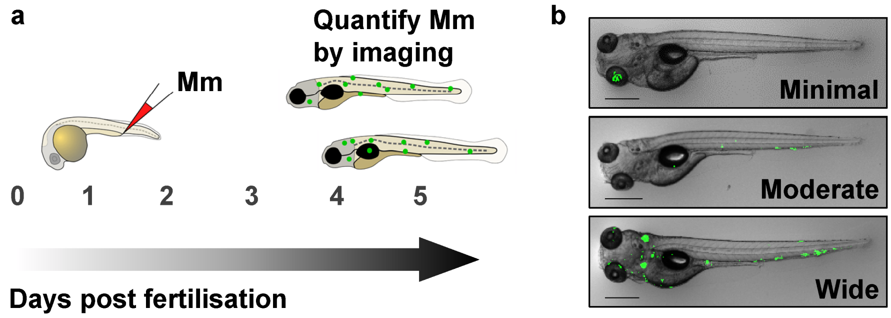

Supplementary Figure S1: Intravenous infection of zebrafish larvae with *Mycobacterium marinum* (Mm).

(a) Schematic diagram of the experimental design. Zebrafish embryos were infected at 28-30 hours post fertilisation by caudal vein injection of Mm expressing mWasabi (green foci), then imaged by stereofluorescence microscopy after four days, to quantify bacterial burden, number of bacterial foci and dissemination. **(b)** Representative images of zebrafish larvae with minimally, moderately and widely disseminated Mm infection. Scale bars, 500 µm.

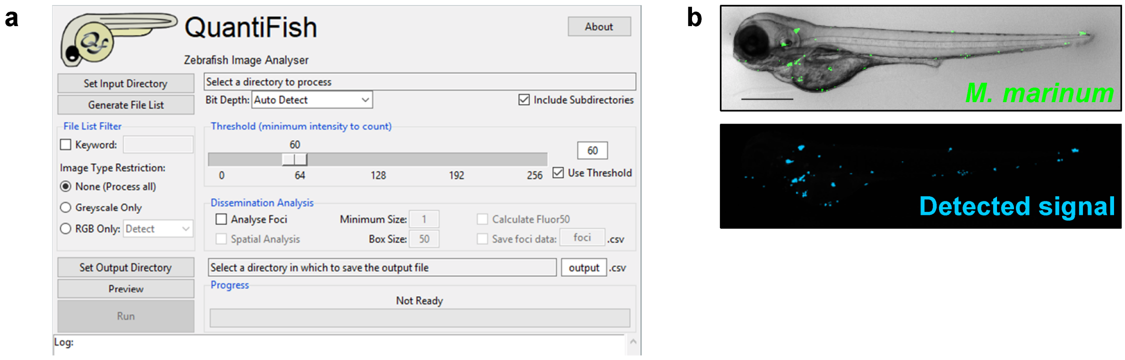

Supplementary Figure S2: Detection and quantitation of fluorescence using QuantiFish.

(a) QuantiFish offers an accessible interface with automatic image bit depth detection, selective file filtering and a previewing pane to visualise detected fluorescence, for rapid quantitation of integrated fluorescence, number of fluorescent foci and four parameters of dissemination. **(b)** Bacterial fluorescence in a zebrafish larva four days post intravenous infection with *Mycobacterium marinum* expressing mWasabi (upper panel), detected using QuantiFish (lower panel). Scale bar, 500 µm.

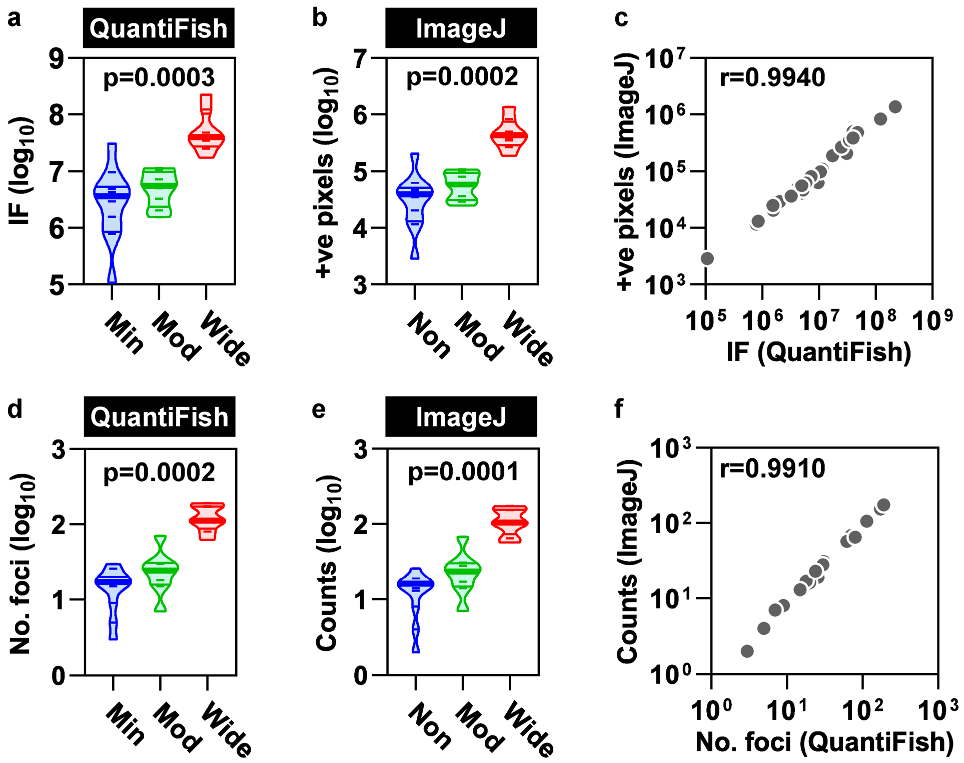

Supplementary Figure S3: Equivalent performance of QuantiFish and ImageJ for quantitation of bacterial burden and counting bacterial foci.

(a) Integrated fluorescence (IF) detected using QuantiFish and **(b)** pixel counts determined using ImageJ, as surrogate measures of total bacterial burden in zebrafish larvae with minimally, moderately and widely disseminated *Mycobacterium marinum* infection (n=11, 8 and 8, respectively). **(c)** The relationship between IF and pixel counts. **(d)** The number of bacterial foci detected using QuantiFish and **(e)** ImageJ and **(f)** the relationship between these measurements. In violin plots **(a, b, d, e)** short dashes represent data points for individual zebrafish larvae and horizontal bars represent median ± IQR. Data points **(c, f)** represent individual zebrafish larvae. p values were derived from Kruskal-Wallis tests with Benjamini, Krieger and Yekutieli correction for multiple comparisons. r values were derived from Spearman rank correlation tests. Data are derived from three independent experiments.

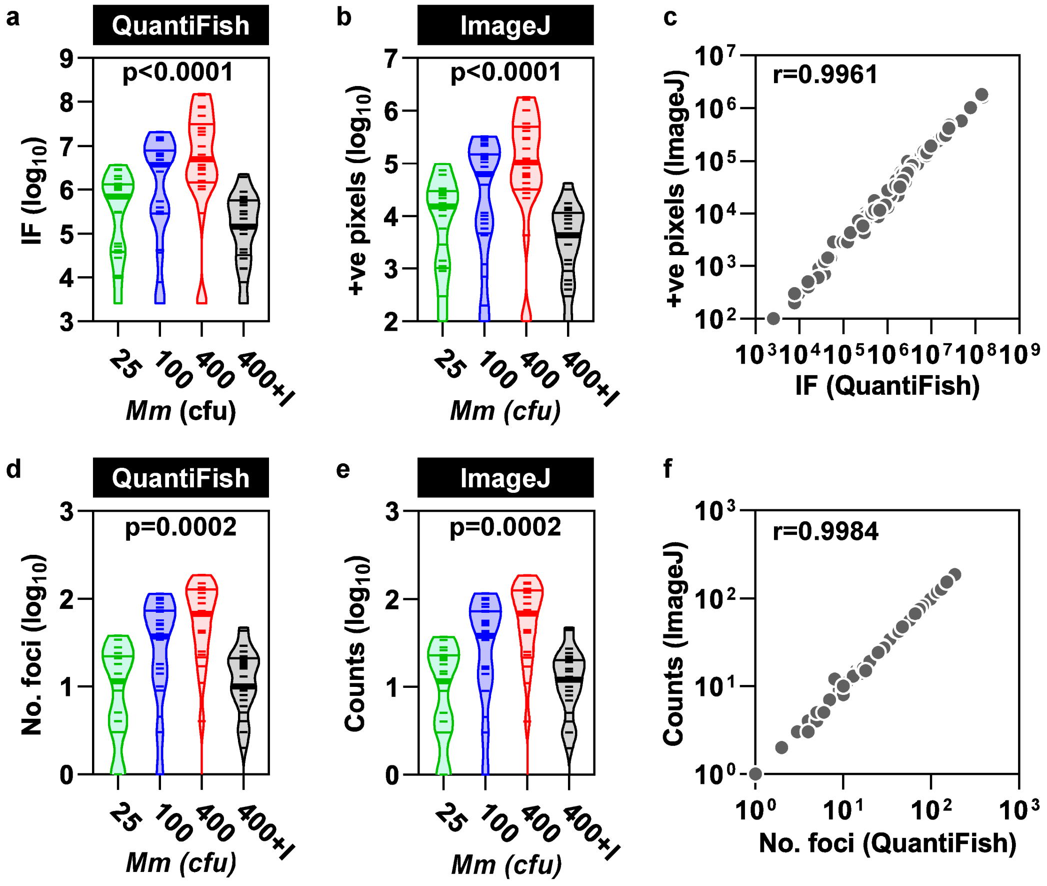

Supplementary Figure S4: Quantitation of bacterial burden and number of bacterial foci in response to a dose titration of intravenous *Mycobacterium marinum* (Mm) infection.

(a) Integrated fluorescence (IF) detected using QuantiFish and **(b)** pixel counts determined using ImageJ, as surrogate measures of total bacterial burden in zebrafish larvae four days after intravenous infection with 25, 100 or 400 cfu Mm ± 400 μM isoniazid (I) (n=24, 28, 22 and 25, respectively). **(c)** The relationship between IF and pixel counts. **(d)** The number of bacterial foci detected using QuantiFish and **(e)** ImageJ and **(f)** the relationship between these measurements. In violin plots **(a, b, d, e)** short dashes represent data points for individual zebrafish larvae and horizontal bars represent median ± IQR. Data points **(c, f)** represent individual zebrafish larvae. p values were derived from Kruskal-Wallis tests with Benjamini, Krieger and Yekutieli correction for multiple comparisons. r values were derived from Spearman rank correlation tests. Data are representative of three independent experiments.

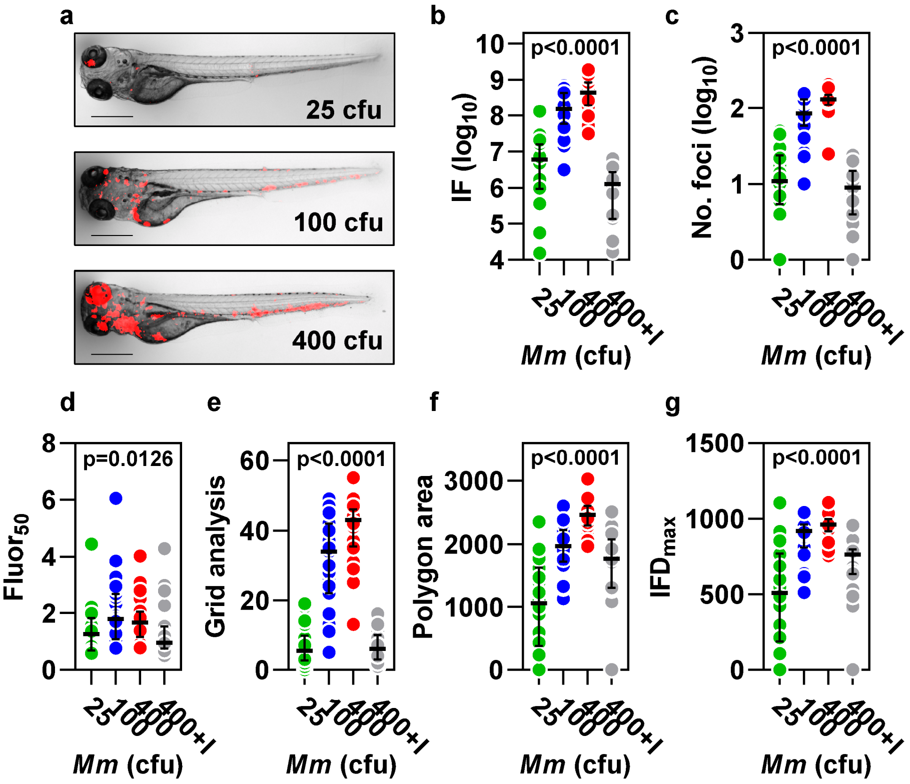

Supplementary Figure S5: Extremely high bacterial burden is associated with a reduction in fluor$_{50}$.

Quantitation of disease severity in zebrafish larvae four days after intravenous infection with 25, 100 or 400 cfu Mm ± 400 µM isoniazid (I) (n=22, 23, 20 and 23, respectively). **(a)** Representative images are shown for each inoculum dose. Scale bars, 500 µm. Existing outcome measures, integrated fluorescence (IF), (**b**) and the number of fluorescent bacterial foci **(c)** and dissemination parameters, fluor$_{50}$ **(d)**, grid analysis **(e)**, polygon area **(f)** and IFD$_{max}$ **(g)** are presented. Data points represent individual zebrafish larvae. Lines and error bars represent median ± IQR. p values were derived from Kruskal-Wallis tests with Benjamini, Krieger and Yekutieli correction for multiple comparisons. Data are representative of two independent experiments.

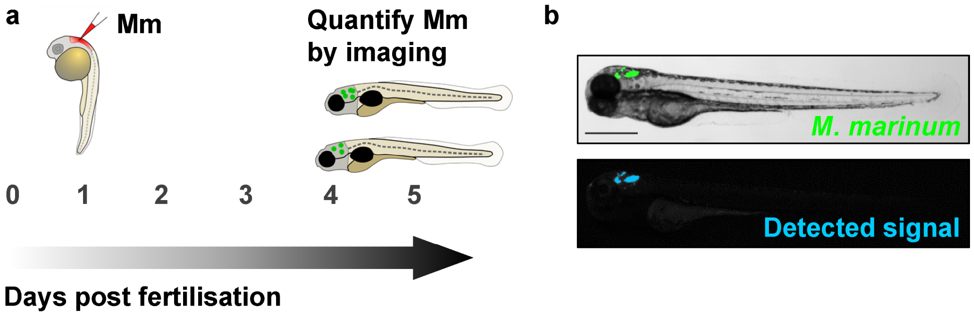

Supplementary Figure S6: Hindbrain ventricle (HBV) infection of zebrafish larvae with *Mycobacterium marinum* (Mm).

(a) Schematic diagram of the experimental design. Zebrafish embryos were infected at 28-30 hours post fertilisation by injection of Mm expressing mWasabi (green foci) into the HBV, then imaged by stereofluorescence microscopy after four days, to quantify bacterial burden, number of bacterial foci and dissemination. **(b)** Representative image of a zebrafish larva with HBV Mm infection (upper panel), detected using QuantiFish (lower panel). Scale bar, 500 µm.

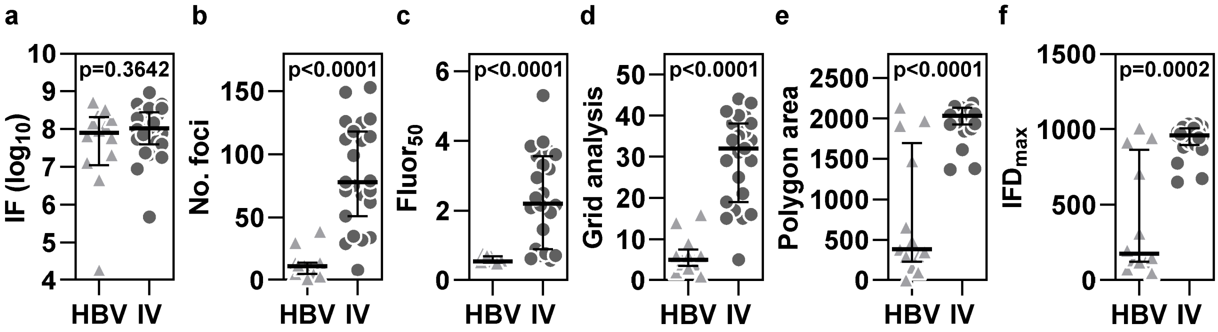

Supplementary Figure S7: Hindbrain ventricle (HBV) *Mycobacterium marinum* (Mm) infection is sometimes distinguished from intravenous infection by fewer fluorescent foci.

Existing outcome measures, integrated fluorescence (IF), a surrogate for total bacterial burden **(a)** and the number of fluorescent bacterial foci **(b)** and dissemination parameters, $fluor_{50}$ **(c)**, grid analysis **(d)**, polygon area **(e)** and IFDmax **(f)** are presented for zebrafish larvae four days after either localised hindbrain ventricle (HBV) infection (n=14) or systemic intravenous (IV) infection (n=23) with 100 cfu Mm. Data points represent individual zebrafish larvae. Lines and error bars represent median ± IQR. p values were derived from Mann-Whitney tests. Data are representative of three independent experiments. See also Fig. 4.
